# Large-Scale Preparation of Silver Nanowire-Based Flexible Transparent Film Heaters by Slot-Die Coating

**DOI:** 10.3390/ma15072634

**Published:** 2022-04-03

**Authors:** Cuilan Liu, Xuyang Zhang, Jiaqi Shan, Zhengliang Li, Xingzhong Guo, Xiaoyu Zhao, Hui Yang

**Affiliations:** 1 State Key Laboratory of Silicon Materials, School of Materials Science and Engineering, Zhejiang University, Hangzhou 310027, China; 3160102802@zju.edu.cn (C.L.); 12026065@zju.edu.cn (X.Z.); zlli@zju.edu.cn (Z.L.); yanghui@zju.edu.cn (H.Y.); 2 Hangzhou Global Scientific and Technological Innovation Center, Zhejiang University, Hangzhou 311200, China; 21626008@zju.edu.cn; 3 Zhejiang Hua Display Optoelectronics Co., Ltd., Jiaxing 314115, China; info@riyngroup.com

**Keywords:** silver nanowires, flexible transparent conductive film, film heater, slot-die coating

## Abstract

Highly flexible silver nanowire-based transparent conductive films (AgNWs TCFs) were large-scale fabricated by slot-die coating AgNWs inks on a flexible polyethylene terephthalate (PET) substrate, and further fabricated into a transparent film heater. Appropriate flow rate, coating speed, and AgNWs concentration allow the construction of the 15 cm × 15 cm AgNW TCFs with a sheet resistance (*R*_s_) of less than 20 Ω/sq, a transmittance (*T*) at 550 nm higher than 95%, and a haze less than 3.5%. The resultant AgNW TCFs heater possesses high uniformity and superior mechanical stability and can reach a Joule heating temperature of 104 °C with a voltage of 12 V. The slot-die coating method has great potential for large-scale production of AgNW based film heaters promisingly used in window defrost and deicer systems.

## 1. Introduction

Transparent conductive films (TCFs) have attracted extensive attention because of their numerous applications, such as solar cells, photovoltaic cells, light-emitting diodes, touch-sensitive screens, and flat-panel displays [1]. As a film that can be electrically conductive and transparent in the visible region, the demand for TCFs continues to increase with the development of electronic products [2]. A variety of materials were identified as candidates for TCFs, such as graphene [3,4], carbon nanotubes (CNTs) [5,6], transparent conductive oxides (TCO) [7,8], polymer conductors [9,10], and metal nanowires [11,12]. Among conductive materials, the most commonly used material is tin-doped indium oxide (ITO) owing to its high electrical conductivity (*R*_s_ < 20 Ω/sq) and optical transmittance (90% at 550 nm) [13,14]. However, ITO has certain limitations for application in next-generation devices:

i. The scarcity of indium and the expensive vapor-phase sputtering process increases its production cost [1];
ii. ITO is brittle and susceptible to failure if the substrate is subjected to bending [15].

Silver-based materials have been important in the scientific field in recent years [16,17]. As reported, silver nanowires (AgNW) are considered to be a favorable material to replace ITO because of their high conductivity, high transparency, suitability for printing, and flexibility [15,18,19]. At present, AgNW-based TCFs can be fabricated through conventional preparation techniques, such as vacuum filtration [20], spin-coating [21,22], drop coating [23], spray-coating [24], Mayer rod coating [25,26], and screen printing [27,28]. However, these conventional preparation methods are difficult when taking into account the conductivity and transmittance of AgNW-based TCFs simultaneously, and it is also difficult to achieve large-scale preparation of TCFs, which limits the application of AgNW-based TCFs. In recent years, slot-die coating has rapidly developed in the preparation of thin films owing to its fast coating speed, high precision, and uniform wet thickness. Slot-die coating can achieve precise preparation of film by extruding and ejecting coating liquid along the gap of the coating die under a certain pressure and flow rate which is transferred to the substrate [29]. Little research on the slot-die coating preparation of AgNW-based TCFs is reported in the present literature.

In this work, we demonstrate the facile fabrication of AgNW TCF heaters with high quality due to slot-die coating. The effects of flow rate, coating speed, and AgNW concentration on the optoelectronic properties of the AgNW TCF were studied, and the heating performance of the resultant AgNW TCF-based heater was also tested. It provides an effective approach to rapidly prepare AgNW TCFs on a large scale.

## 2. Materials and Methods

### 2.1. Preparation of AgNW TCF Heaters

AgNW dispersion with an average diameter of 45 nm and an average length of 40 μm was purchased from Zhejiang Kechuang Advanced Materials Technology Co. Ltd. (Hangzhou, China), (hydroxypropyl)methylcellulose (HPMC) was obtained from Sinopharm Chemical Reagent Co. Ltd. (Shanghai, China), and Zonyl FSO-100 was obtained from DuPont Co. Ltd (Wilmington, NC, USA). In the experiment, AgNW (10 mg·mL^−1^), HPMC (8 mg·mL^−1^), and FSO-100 (1 mg·mL^−1^) were added to 10 mL deionized water and stirred for 1 h to obtain the AgNW conductive ink. An automatic slot-die coating machine (PE Coater-S300, Hunan NanoUp Electronics Technology Co., Ltd. (Changsha, China)) was utilized to coat AgNW composite ink on PET substrates. After filling the injector with the ink, the AgNWs TCFs were fabricated by slot-die coating with adjustable operating conditions including the slot-die coating speed of 3–8 mm·s^−1^, the flow rate of AgNW conductive ink of 0.5–2.5 mL·min^−1^ and the PET substrate temperature of 45 °C. On the basis of as-prepared AgNW TCF, the AgNW TCF-based heater was constructed. The silver conductive paint lines were printed on the two terminal sides of AgNW TCF by screen printing, which acted as bonding materials and electrodes for electrical contact.

### 2.2. Characterization

Scanning electron microscopy (SEM) of the AgNW TCF was observed by a SU8010 high-resolution microscope (Tokyo, Japan) with an accelerating voltage of 3 kV. The transmittance spectra were performed with an ultraviolet-visible spectrometer (UV-2600i, Shimadzu, Kyoto, Japan)). The haze of the AgNW TCF was tested using a haze tester (WGT–S, INESA, Shanghai, China). The sheet resistance of the AgNW TCF was measured by a four-point probe sheet resistance meter (RTS-9, Guangzhou four-point probe technology, Guangzhou, China). The roughness of the film surface was achieved by atomic force microscopy (AFM, Dimension Icon, Bruker, MA, USA). The viscosity could be measured by a viscometer (DV-II + Pro, Brookfield, MA, USA). The contact angle was obtained by using a video-based contact angle measuring device (OCA 20, Dataphysics, Stuttgart, Germany). The bending test was finished by a self-made slipway and the resistance changes were monitored by a lab-made digital multimeter. The DC voltage supply and current measurement were provided by a Precise S100 source meter (Wuhan, China). The heating temperature of the AgNW TCF transparent heater was monitored using a CENTER 309 temperature detector (Taiwan, China) adhered to the middle point of the bottom of the heater and continuously monitored using data collection software (TestLink SE-309, Yuanhengtong technology Co. Ltd., Shenzhen, China). The infrared images were recorded using a DL 700E + infrared thermal imager (Hangzhou, China).

## 3. Results and Discussion

### 3.1. Construction of AgNW TCF by Slot-Die Coating

**Figure 1:**
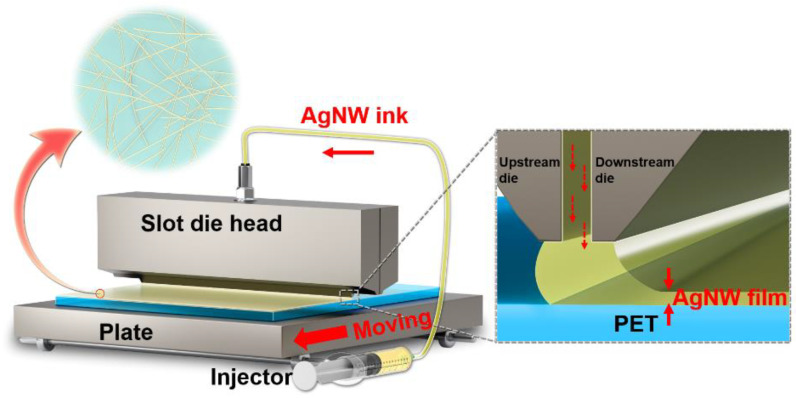
Schematic diagram of slot-die coating process of AgNW TCFs.

An AgNW TCF can be facilely fabricated by slot-die coating, as shown in Figure 1. In the coating process, an AgNW ink is pumped from the injector with a set flow rate into the slot-die head, and then the coating AgNW ink forms a meniscus to the PET substrate which is moved under the slot-die head. The slot-die head is placed close to the moving substrate so that once the AgNW ink exits the slot-die head the AgNW ink will enter the gap between the head and the substrate, and then enter one of two constrained channels in the upstream and downstream directions. Since the slot-die head is effectively fixed and the substrate moves at a set speed, this results in a varying flow rate between the bottom and top of the channel. The flow rate increases linearly with the channel profile, and the overall profile of solution flow between the upstream and downstream lips will vary. After the coating, an AgNW TCF can be prepared on the PET substrate. The AgNWs pile up and lap under the action of gravity to form a conductive network, providing channels for carrier transport. In the coating process, the wet film thickness is mainly determined by the slot-die coating speed and ink flow rate at a set coating width.

In this work, the hybrid AgNW conductive ink consists of conductive materials, binder, additive, and solvent. AgNW dispersion is used as the conductive material to form a percolation network at a low loading density. Distilled water is chosen as a solvent due to its environmental friendliness and low cost. HPMC is a water-soluble viscoelastic polymer that can help enhance the adhesion between AgNWs and PET. AgNWs are attached to the substrate only by gravity with weak adhesion, without HPMC. The addition of HPMC can cover and protect the AgNWs. As Table S1 shows, its viscosity is much higher than water, and the AgNW ink with HPMC exhibits a relatively high viscosity of 11.4 cps, enhancing its adhesion to the substrate. Zonyl FSO-100, a nonionic and water-soluble fluorosurfactant is selected as the additive because it can decrease the surface tension of the water-based ink, and thus promote substrate wettability. Figure S1 shows the contact angles between different materials and the PET substrate, which can reflect their surface tensions. The greater the contact angle, the greater the surface tension. FSO-100 has a low surface tension on the PET compared to water, and the AgNW ink with FSO-100 exhibits a contact angle of 50.7°.

In general, the electrical and optical properties of the AgNW TCFs can be improved by optimizing conditions such as the distance between the slot-die head and the flexible substrate, the flow rate, the slot-die coating speed, and the temperature of the PET substrate. Among these parameters, the flow rate, the slot-die coating speed, and the concentration of AgNWs have great influences on the properties of the AgNW TCFs. They can largely determine the density of AgNWs on PET, thus affecting the performance of AgNW TCF. A high density of AgNWs can decrease the *R*_s_ of the film but it will also decrease the transmittance and increase the haze, which will adversely affect its application for optoelectronic devices. In order to balance its performance, it is important to select proper parameters. During the experiment, the distance between the slot-die and the PET substrate is fixed to 500 μm, and the shim is 100 μm. Meanwhile, the performance of the film prepared with ink without AgNWs is tested to eliminate the influence of the HPMC and FSO-100. This film is nonconductive and performs better in transmittance. With the PET as a blank substrate, the transmittance of this thin film is shown in Figure S2. Its transmittance reaches a value of more than 100%, resulting from the anti-reflection function of HPMC.

### 3.2. Optoelectric Performance Optimization of Slot-Die Coated AgNW TCFs

**Figure 2:**
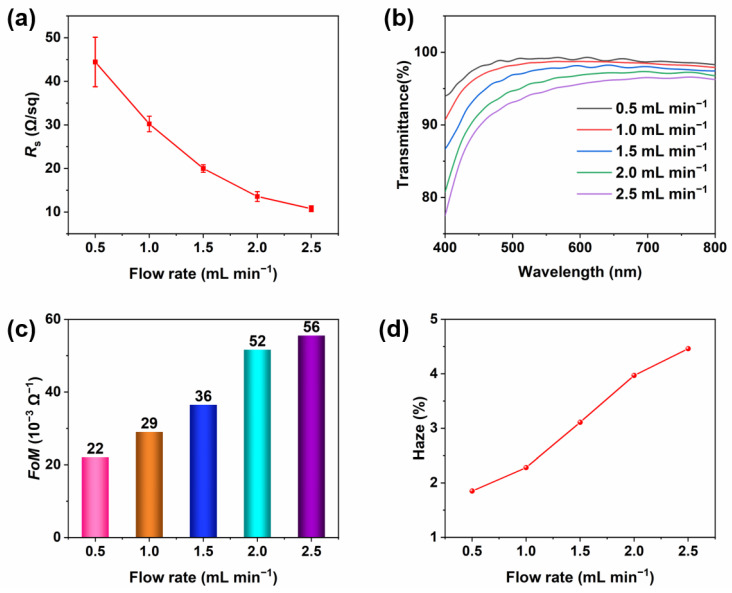
Optoelectronic properties of the AgNW film produced at different flow rates: (**a**) the *R*_s_ of the AgNW films; (**b**) the transmittance of the AgNW films from 400 nm to 800 nm; (**c**) the *FoM*; (**d**) the haze values of the AgNW films.

Large-scale AgNW TCFs were fabricated by the slot-die coating process at room temperature under ambient air and the preparation conditions, including flow rate, coating speed, and AgNW concentration, were adjusted to optimize the optoelectronic properties of the AgNW TCF. Figure 2 shows the difference in the properties of the AgNW film produced by varying the flow rate. To facilitate the comparison, the mean values and standard deviations of the *R*_s_ of these films are calculated and shown in Figure 2a. As the flow rate increases, the mean value of *R*_s_ decreases from 44.4 to 10.8 Ω/sq with the increasing density of the AgNWs. To evaluate the uniformity of these films, which is difficult to maintain especially when coating a large area, the standard deviation is calculated by taking samples from different locations of the film. The standard deviations of the *R*_s_ were 5.7, 1.8, 0.9, 1.1, and 0.7 Ω/sq at flow rates of 0.5, 1.0, 1.5, 2.0, and 2.5 mL min^−1^, respectively. When the flow rate is more than 2 mL min^−1^, there is less of a decrease in the standard deviations of the *R*_s_. At a high flow rate, the electron charge can be stably transferred and the surface uniformity is optimized. Figure 2b shows the transmittances of the TCFs at different flow rates. As the flow rate increases, the transmittance decreases continuously because the higher content of AgNWs prevents visible light from passing through the voids of the AgNW conductive network. To evaluate the performance of the AgNW TCF, a figure of merit (*FoM)* value is often introduced to quantify its properties. The higher the *FoM*, the better the optoelectronic performance of the AgNW TCF. There are two kinds of calculation formulas for the *FoM*. The first one comes from the relationship between *T* and *R*_s_, as given by [30]:

(1) $$T={\left(1+\frac{Z_{0}\sigma_{\mathrm{opt}}}{2R_{s}\sigma_{\mathrm{DC}}}\right)}^{-2}$$

where *T* denotes the transmittance at 550 nm, *R*_s_ represents the sheet resistance, *Z*_0_ is the free space impedance (377 Ω), and *σ*_DC_ and *σ*_opt_ are the electronic and optical conductivity, respectively. The *FoM* calculation formula is defined as the ratio of *σ*_DC_ and *σ*_opt_. Thus, the *FoM* calculation formula is defined as follows:

(2) $$\frac{1}{R_{s}}=FoM\cdot \frac{\left(T^{-0.5}-1\right)}{188.5\;\Omega }$$

However, this calculation formula is not correct in this work as part of the ink has the function of anti-reflection, the *FoM* of a TCF with relatively high *R*_s_ and ultra-high transmittance can reach an ultra-high value, even infinity when the transmittance is close to 100%. Therefore, the Haacke index is chosen as follows, which is a figure of merit for transparent electrode materials:

(3) $$\emptyset_{TC}=\frac{T^{10}}{R_{s}}$$

where *T* is the transmittance at 550 nm and *R*_s_ is the sheet resistance of the TCF. The *FoM*s of the AgNW TCFs at different flow rates are studied in Figure 2c. With the increase in the flow rate, the *FoM* increases gradually from 22 × 10^−3^ Ω^−1^ to 56 × 10^−3^ Ω^−1^. Figure 2d shows the haze values of the AgNW films. Here, haze is defined as the percentage of transmitted light passing through the film that deviates by more than 2.5° from the incident beam by forward scattering [31]:

(4) $$Haze=\frac{{\left(I_{s}\right)}_{2.5{}^{\circ}-90{}^{\circ}}}{I_{d}+{\left(I_{s}\right)}_{f}}$$

where *I*_d_ is the light flux transmitted directly and (*I*_s_)_f_ is the flux undergoing forward scattering, i.e., the scattered intensity between 0° and 90°. The haze value increases gradually from 1.85% to 4.46% as the flow rate increases. In this work, the AgNW TCF with an *R*_s_ of below 20 Ω/sq, the *R*_s_ standard deviations of below 1 Ω/sq, a transmittance at 550 nm of over 95%, an *FoM* of over 35 × 10^−3^ Ω^−1,^ and a haze of below 3.5%, are expected to be obtained after optimization. To determine the most suitable flow rate, the comparison of these different parameters is represented in Figure S3, with the above-mentioned criteria being considered. It is worth mentioning that these criteria are subject to discussion, which is only used to compare the properties between different samples and select a good TCF. Among these flow rates, only 1.5 mL·min^−1^ can meet all the goals, thus being the best flow rate at which the AgNW TCF could show better performance.

**Figure 3:**
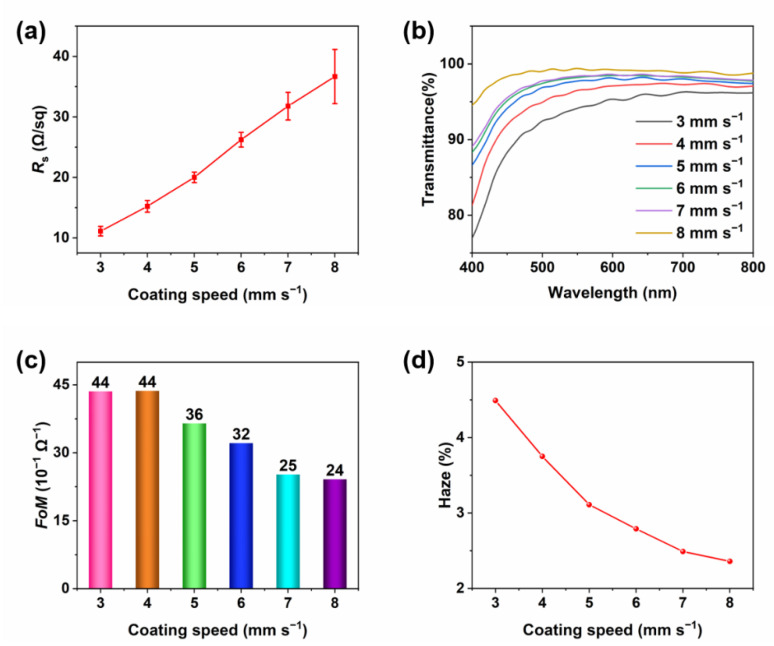
Optoelectronic properties of the AgNW film produced at different coating speeds: (**a**) the *R*_s_ of the AgNW films; (**b**) the transmittance of the AgNW films from 400 nm to 800 nm; (**c**) the *FoM*; (**d**) the haze values of the AgNW films.

Based on the determined flow rate at 1.5 mL·min^−1^, the performance of the AgNW TCFs at different slot-die coating speeds is observed in Figure 3. The mean values and standard deviations of the *R*_s_ of these films are calculated and shown in Figure 3a. With the increase in the slot-die coating speed, the *R*_s_ increases, resulting from the decreased density of the AgNWs. Overall, the standard deviations of *R*_s_ are relatively low, indicating great uniformity. Figure 3b shows the transmittances of the TCFs at different slot-die coating speeds. At a certain flow rate, a higher coating speed means a thinner AgNW film, thus the density of the AgNWs is lower and the transmittance will be higher. Due to the small adjustment of the coating speeds in the experiment and the non-uniformity of the films, the transmittance of some films is very close in some wavelengths. As Figure 3c shows, the *FoM* decreases from 44 × 10^−3^ to 24 × 10^−3^ Ω^−1^ with the increase in coating speed. In Figure 3d, the haze value decreases gradually from 4.49% to 2.36% as the coating speed increases, resulting in the density reduction of the AgNWs. In Figure S4, 5 mm·s^−1^ is chosen as the best coating speed to obtain an AgNW TCF. Given all these results, a slot-die coating process with a flow rate of 1.5 mL·min^−1^ and a slot-die coating speed of 5 mm·s^−1^ is considered the best fabrication process.

**Figure 4:**
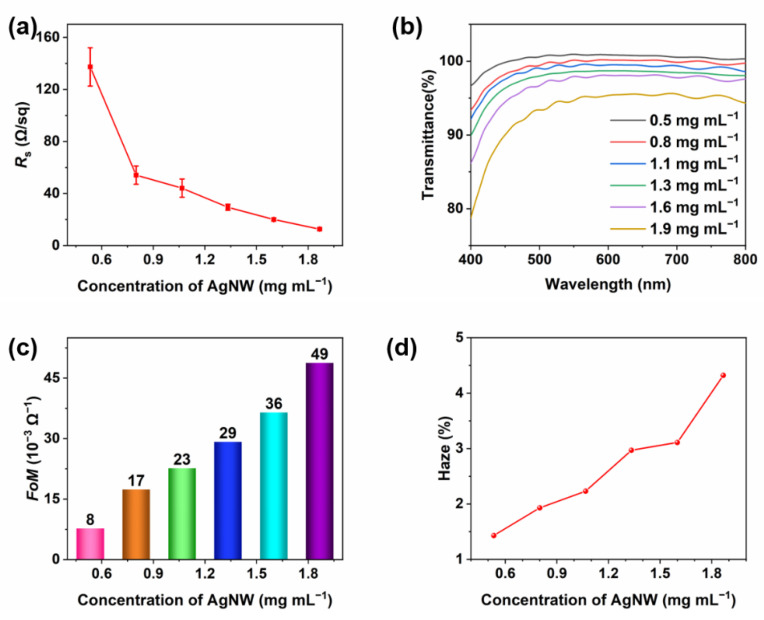
Optoelectronic properties of the AgNW film produced at different AgNW concentrations: (**a**) the *R*_s_ of the AgNW films; (**b**) the transmittance of the AgNW films from 400 to 800 nm; (**c**) the *FoM*; (**d**) the haze values of the AgNW films.

The concentration of the AgNWs can greatly affect the properties of the TCFs. In general, an AgNW TCF with a higher concentration of the AgNWs can provide more conductive pathways and block the passage of light. Therefore, it is necessary to obtain a suitable AgNW concentration. As shown in Figure 4, the properties of the AgNW film change significantly at different concentrations of AgNWs. As a result, the *R*_s_ and transmittance of the films decrease continuously with the increase in concentration, indicating that more conductive paths are formed and the ability of the light of each wavelength to pass through the films decreases with the increase in the density of AgNWs. At a low concentration of 0.5 mg·mL^−1^, the *R*_s_ of AgNW TCF is 137.3 Ω/sq, which is far higher than that with other concentrations, and far from the requirements of practical application. This is because very low-density nanowires cannot form adequate conductive pathways. It is worth mentioning that this also reduces the loss of transmittance, especially with the anti-reflection effect of other components in the ink, which show a transmittance of more than 100% in some wavelengths. Meanwhile, they have poor uniformity with a standard deviation of *R*_s_ at 14.7 Ω/sq. When the concentration increases from 0.8 to 1.9 mg·mL^−1^, the mean values and standard deviations of *R*_s_, and the transmittance of the films decrease slowly. It can be seen from Figure 4c that the *FoM* values of the AgNW TCFs increase with the increase in concentration. Figure 4d shows the haze values of the AgNW films at different AgNWs concentrations. A higher concentration of AgNWs will result in a greater haze of the film due to the light scattering of the AgNWs.

**Figure 5:**
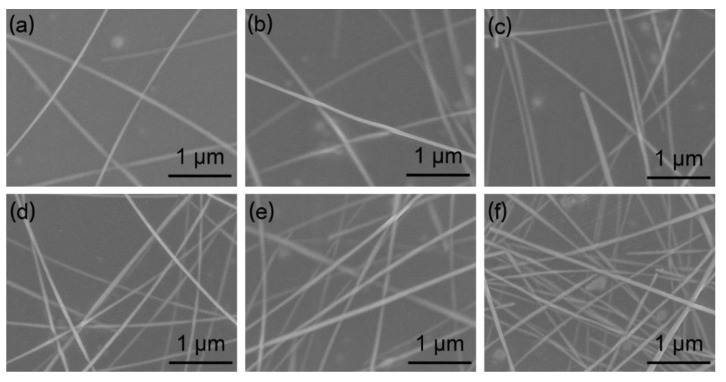
SEM morphology of AgNW TCFs prepared at different AgNW concentrations: (**a**) 0.5, (**b**) 0.8, (**c**) 1.1, (**d**) 1.3, (**e**) 1.6, and (**f**) 1.9 mg·mL^−1^.

Figure 5 shows the scanning electron microscope (SEM) morphology of the AgNW TCFs prepared at different concentrations. As shown in Figure 5, the distribution of the AgNWs is very sparse at a low concentration of 0.5 mg·mL^−1^ (Figure 5a), and there are only a few contact points between the AgNWs. As a result, there are only a small number of conductive pathways in the network, which are consistent with the previous results. As the concentration increases from 0.8 to 1.9 mg mL^−1^, this situation is gradually improved. As the density of the AgNWs increases, more points of contact are formed to construct a larger number of conductive pathways in the network, providing more channels for carrier transport. As shown in Figure S5, 1.6 mg·mL^−1^ is determined as the final concentration.

**Figure 6:**
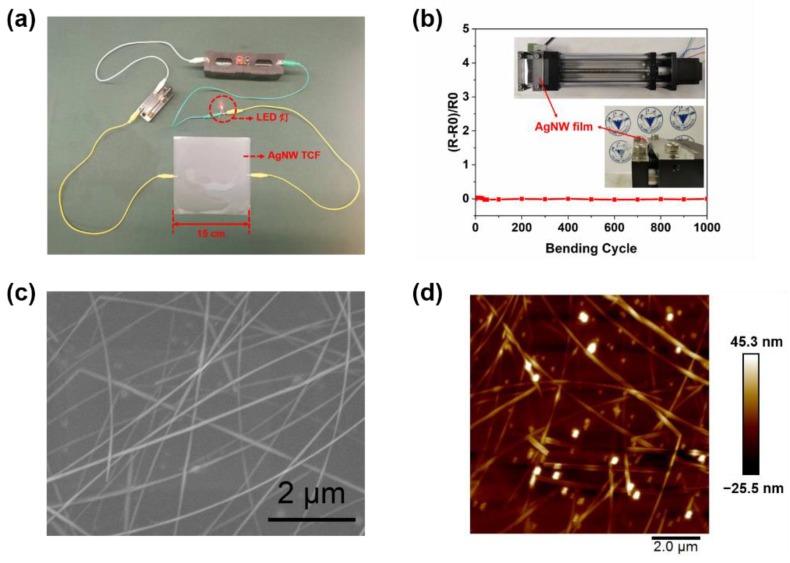
(**a**) The photograph of AgNW TCF (15 cm × 15 cm) connected in series with a power supply, LED, switch, and wires. (**b**) Variations in resistance of the optimized AgNW TCF during repeated bending test. (**c**) SEM morphology of the optimized AgNW TCF (**d**) AFM image of the optimized AgNW TCF.

Based on all of the above data, a high quality AgNW TCF (15 cm × 15 cm) can be fabricated at a flow rate of 1.5 mL·min^−1^, a coating speed of 5 mm·s^−1^, and an AgNW concentration of 1.6 mg·mL^−1^, shown in Figure 6a. The LED light is lit, which directly proves the conductivity of the film. Under optimized preparation conditions, AgNW TCF exhibits excellent performances (*R*_s_ ≤ 20 Ω/sq, *T* at 550 nm ≥ 95%, the standard deviation of *R*_s_ ≤ 1 Ω/sq, *FoM* ≥ 35 × 10^−3^ Ω^−1^, haziness ≤ 3.5%). Compared with other similar studies, the AgNW TCF in our work has excellent photoelectric properties, as shown in Table 1.

Moreover, the resultant AgNW TCF has good flexibility. A film (5 cm × 5 cm) is cut out for the bending test and the resistance of the film is almost unchanged after 1000 bendings, as shown in Figure 6b, indicating its potential application in flexible wearable devices. However, the environmental stability of the AgNW TCF needs to be improved. The resistance of the film to ambient air (25 ℃, 38%) increases in 60 h, which results from the oxidation of AgNW, as shown in Figure S6. AgNWs are well-dispersed on the PET substrate, as shown in Figure 6c. Figure 6d shows the tapping-mod AFM image of the optimized AgNW TCF. It clearly shows that the AgNW TCF has an RMS roughness value of 9.07 nm.

### 3.3. Joule Heating Performance of AgNW TCF Based Heaters

**Figure 7:**
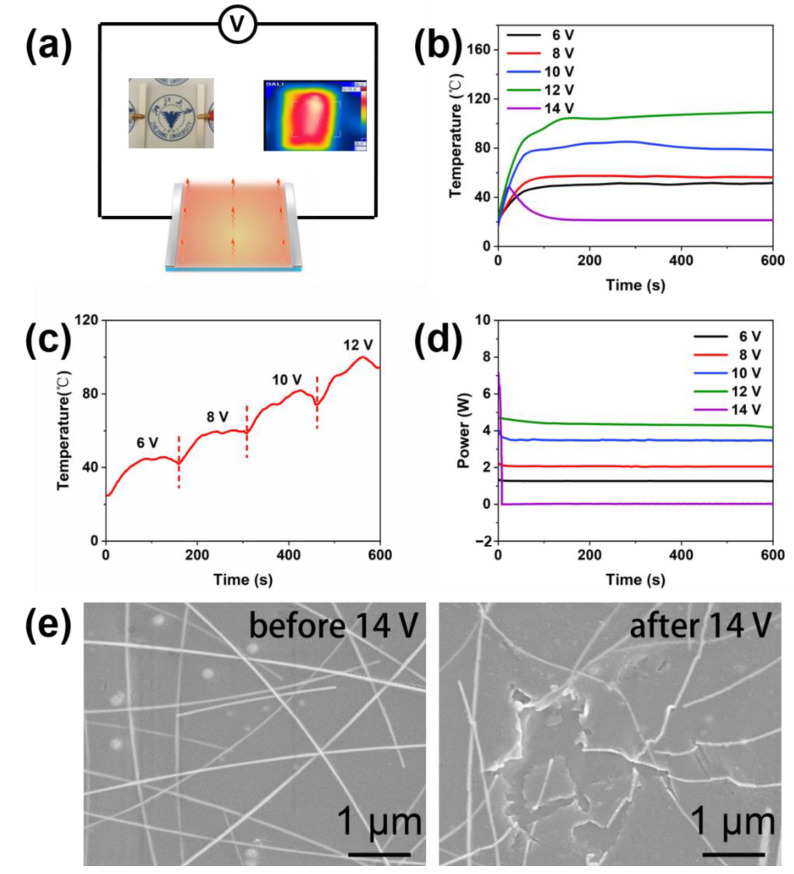
(**a**) The schematic illustration and the infrared image of the AgNW film heater (5 cm × 5 cm). (**b**,**c**) Temperature-time profile at different applied voltages. (**d**) Power-time profile at different applied voltages. (**e**) SEM images of the AgNW heater before and after an applied voltage of 14 V.

On the basis of as-prepared high-quality AgNW TCFs, a transparent flexible heater is prepared. Figure 7a shows the schematic of the AgNW TCF-based heater. Two silvery lines at the edges of the AgNW thin film are the silver current collectors. When an electrical current flows across the AgNW transparent heater, it generates heat thanks to the Joule effect. Infrared photography of the film heater at a constant applied voltage clearly reveals that the temperature distribution on the film area is uniform, indicating that the AgNW film is uniformly formed. To verify its possibilities in transparent heaters, the temperature and power of the AgNW heater are monitored at different applied voltages in 600 s, shown in Figure 7b–d. Driven by a specific voltage, the temperature of the AgNW heater increases rapidly from room temperature to a high-temperature value and then keeps almost constant. The AgNW heater can be heated to the equilibrated temperature of approximately 50, 57, 84, and 104 °C driven by 6, 8, 10, and 12 V, respectively, within less than approximately 150 s, indicating the fast response of the conductive AgNW heater to the applied voltage. The heating power can reach 1.3, 2.1, 3.6, and 4.6 W at 6, 8, 10, and 12 V, respectively. It is interesting to observe the temperature higher than 100 °C at 12 V, which is exactly the general vehicle charger output voltage, indicating its potential as a car window defroster. However, the temperature of the heating film is unstable with a line fluctuation at 10 and 12 V (Figure 7b). At 14 V, its temperature drops to room temperature in 150 s, and its heating power drops to 0 W within 10 s. Figure 7e shows the morphology of the AgNW heater before and after being used at 14 V. It is obvious that the nanowires in the heater break at 14 V, and the broken AgNWs destroy the original conductive network. To prevent the AgNW heater from failure, the applied voltage should not exceed 12 V.

## 4. Conclusions

In summary, an AgNW TCF was facilely fabricated by slot-die coating AgNW ink on a PET substrate, and the preparation conditions including flow rate, coating speed, and AgNW concentration were adjusted to optimize the optoelectronic properties of the AgNW TCF. An AgNW flexible film (15 cm × 15 cm) with high uniformity (the standard deviation of *R*_s_ ≤ 1 Ω/sq) and good electrical (*R*_s_ ≤ 20 Ω/sq) and optical (*T* ≥ 95% at 550 nm, haziness ≤ 3.5%) properties was produced at a flow rate of 1.5 mL·min^−1^, a coating speed of 5 mm·s^−1^ and an AgNW concentration of 1.6 mg·mL^−1^. The resultant AgNW transparent heater exhibited good thermal properties and could reach a Joule heating temperature of 104 °C with a voltage of 12 V. This approach will promote the development of large-scale AgNW TCF heaters for applications in window defrosting and deicer systems.

## Figures and Tables

**Table 1:** Optoelectronic properties of the optimized AgNW TCF compared with other similar studies.

Material	Method	Mean Value of *R_s_* (Ω/sq)	*T* at 550 nm (%)	The Standard Deviation of *R_s_* (Ω/sq)	Reference
AgNW	Slot-die	20	96.9	0.9	Our work
AgNW		30.9	86.0	11.4	[2]
AgNW		32	86.2	/	[29]
AgNW		30	89	/	[32]
Carbon nanotube (CNT)/AgNW		102	87.03	/	[33]

## Data Availability

The data presented in this study are available on request from the corresponding author.

## Supplementary Materials

The following supporting information can be downloaded at: https://www.mdpi.com/article/10.3390/ma15072634/s1, Table S1: The viscosity of some materials in AgNW ink; Figure S1: The contact angles between PET and pure water (a), Zonyl FSO-100 (b), AgNW ink (c); Figure S2: The transmittance of the film fabricated by an ink without AgNWs; Figure S3: Comparison of AgNW TCFs fabricated with different flow rates; Figure S4: Comparison of AgNW TCFs fabricated with different coating speeds; Figure S5: Comparison of AgNW TCFs fabricated with different concentrations of AgNWs; Figure S6: The resistance change of the AgNW TCF in presence of ambient air (25 ℃, 38%).
