# Supplementary material for: Large-Scale Preparation of Silver Nanowire-Based Flexible Transparent Film Heaters by Slot-Die Coating
Source: Materials (Basel). 2022 Apr 3;15(7):2634. doi: 10.3390/ma15072634 (PMC9000623; doi:10.3390/ma15072634)
Supplement: Supplementary file 1 [file materials-15-02634-s001.zip › materials-1658037-supplementary.pdf]

materials

Supplementary Materials: Large-Scale Preparation of Silver Nanowire-Based Flexible Transparent Film Heaters by Slot-Die Coating

Cuilan Liu [1], Xuyang Zhang [1], Jiaqi Shan [2], Zhengliang Li [1], Xingzhong Guo [1,2,*], Xiaoyu Zhao [3] and Hui Yang [1,2]

[1] State Key Laboratory of Silicon Materials, School of Materials Science and Engineering, Zhejiang University, Hangzhou 310027, China; 3160102802@zju.edu.cn (C.L.); 12026065@zju.edu.cn (X.Z.); zlli@zju.edu.cn (Z.L.); yanghui@zju.edu.cn (H.Y.)
[2] Hangzhou Global Scientific and Technological Innovation Center, Zhejiang University, Hangzhou 311200, China; 21626008@zju.edu.cn
[3] Zhejiang Hua Display Optoelectronics Co., Ltd., Jiaxing 314115, China; info@riyngroup.com
* Correspondence: msewj01@zju.edu.cn; Tel.: +86-571-87953313

The slot die production of large scale silver nanowire films for flexible transparent film heaters

Table S1. The viscosity of some materials in AgNW ink.

Sample	Viscosity(cps)
Water	1.0
HMPC (8 mg/ml)	260.3
AgNW ink	11.4

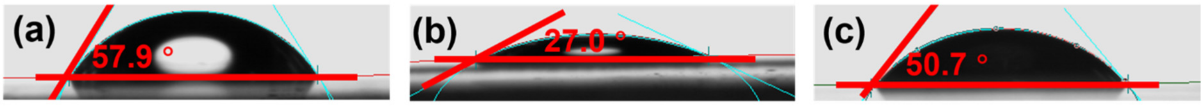

Figure S1. The contact angles between PET and pure water (a), Zonyl FSO-100 (b),AgNW ink (c)

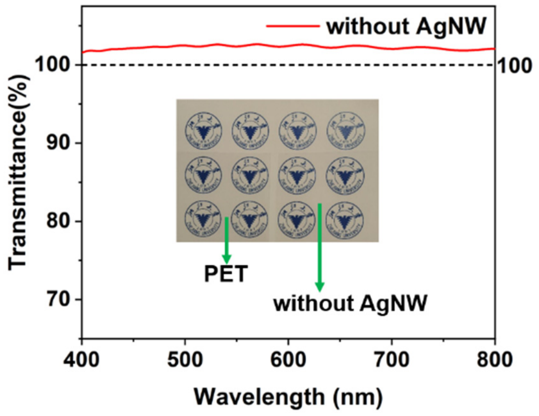

Figure S2. The transmittance of the film fabricated by an ink without AgNWs.

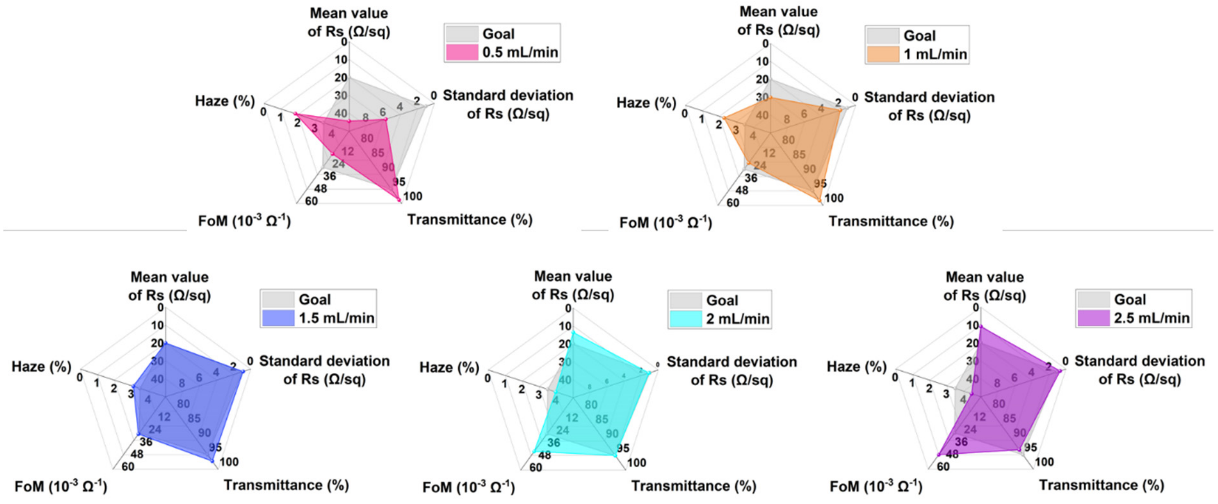

Figure S3. Comparison of AgNW TCFs fabricated with different flow rates. This figure shows the gap between the sample performance and the goal performance.

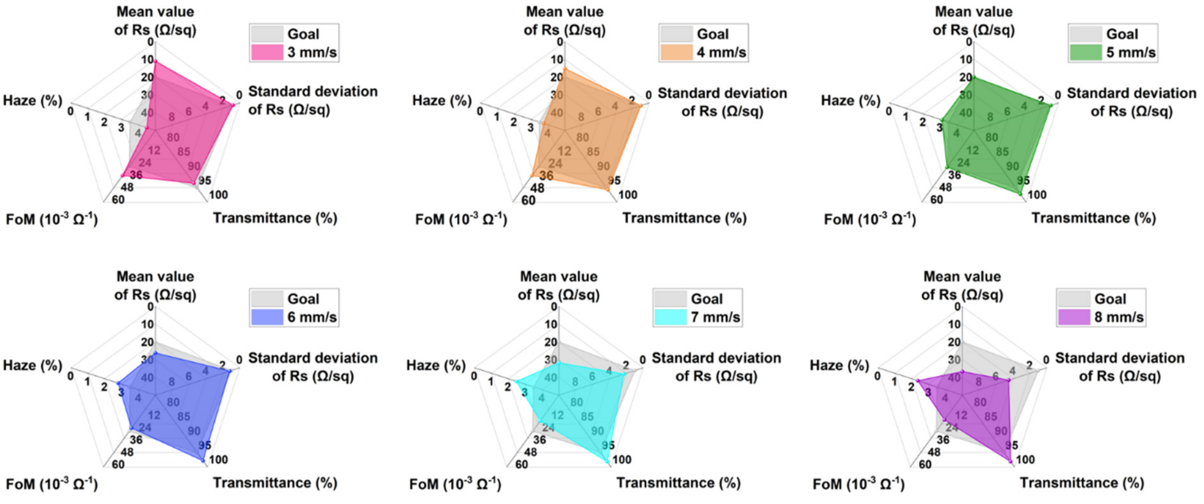

Figure S4. Comparison of AgNW TCFs fabricated with different coating speeds. This figure shows the gap between the sample performance and the goal performance.

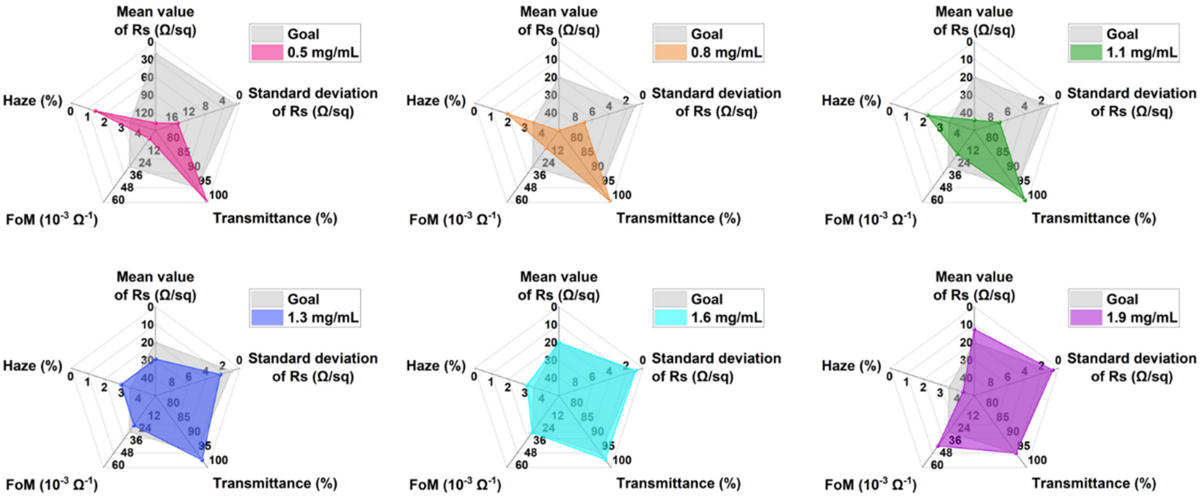

Figure S5. Comparison of AgNW TCFs fabricated with different concentrations of AgNWs. This figure shows the gap between the sample performance and the goal performance.

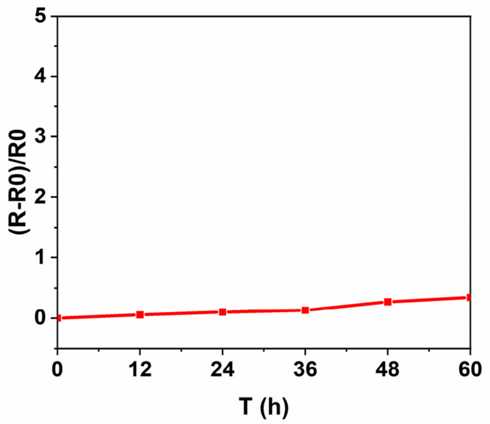

Figure S6. The resistance change of the AgNW TCF at ambient air (25 °C, 38%).

© 2022 by the authors. Licensee MDPI, Basel, Switzerland. This article is an open access article distributed under the terms and conditions of the Creative Commons Attribution (CC BY) license (https://creativecommons.org/licenses/by/4.0/).
